# To the Medical Profession

**Published:** 1851-09

**Authors:** John Evans

**Affiliations:** No. 108 Randolph St., Chicago


### ARTICLE III.

### TO THE MEDICAL PROFESSION OF ILLINOIS.

Having been selected by the Chairman, Prof. T. Reyburn, of St. Louis, as an associate member of the Committee of the American Medical Association on the Epidemic Diseases of Missouri, Illinois, Iowa and Wisconsin, and the consideration of the subject for the State of Illinois having been assigned to me, I would solicit reports from the physicians of the state of any and all epidemics that have prevailed within their respective regions of country from the commencement of the year 1849 up to the close of 1851. Information furnished shall be duly credited in the report.

JOHN EVANS.

No. 108 Randolph St., Chicago.

